# Exosomes derived from stem cells of human deciduous exfoliated teeth inhibit angiogenesis in vivo and in vitro via the transfer of miR-100-5p and miR-1246

**DOI:** 10.1186/s13287-022-02764-9

**Published:** 2022-03-03

**Authors:** Panpan Liu, Qun Zhang, Jun Mi, Shuangshuang Wang, Qiuping Xu, Dexuan Zhuang, Wenqian Chen, Chang Liu, Liwei Zhang, Jing Guo, Xunwei Wu

**Affiliations:** 1 Department of Tissue Engineering and Regeneration, School and Hospital of Stomatology, Cheeloo College of Medicine, Shandong University & Shandong Key Laboratory of Oral Tissue Regeneration & Shandong Engineering Laboratory for Dental Materials and Oral Tissue Regeneration, No.44-1 Wenhua Road West, Jinan, Shandong China; 2 Department of Pediatrics Dentistry and Preventive Dentistry, School and Hospital of Stomatology, Cheeloo College of Medicine, Shandong University & Shandong Key Laboratory of Oral Tissue Regeneration & Shandong Engineering Laboratory for Dental Materials and Oral Tissue Regeneration, No.44-1 Wenhua Road West, Jinan, Shandong China; 3 Department of Orthodontics, School and Hospital of Stomatology, Cheeloo College of Medicine, Shandong University & Shandong Key Laboratory of Oral Tissue Regeneration & Shandong Engineering Laboratory for Dental Materials and Oral Tissue Regeneration, No.44-1 Wenhua Road West, Jinan, Shandong China; 4 Cutaneous Biology Research Center, Massachusetts General Hospital, Harvard Medical School, Boston, MA USA; 5 Savaid Stomatology School of Hangzhou Medical College, Ningbo Stomatology Hospital, Ningbo, China

**Keywords:** Exosome, Stem cells of human deciduous exfoliated teeth, Angiogenesis, miR-100-5p, miR-1246

## Abstract

**Background:**

Anti-angiogenic therapy has been shown to be a promising strategy for anti-tumor treatment. Increasing evidence indicates that tumor angiogenesis is affected by exosomes that are secreted by mesenchymal stem cells (MSCs), but whether exosomes derived from MSCs suppress or promote angiogenesis remain paradoxical. The purpose of this study focused on understanding the potential role of exosomes derived from stem cells of human deciduous exfoliated teeth (SHED-Exos) in regulating angiogenesis and the underlying molecular mechanism.

**Methods:**

Exosomes were isolated from supernatants of SHED cells using an exosome purification kit and were characterized by transmission electron microscopy, nanoparticle tracking analysis and western blot analysis. Cell Counting Kit-8, flow cytometric assays, western blots, wound healing and transwell migration assays were performed to characterize the roles of SHED-Exos on cell proliferation, apoptosis and migration of human umbilical vein endothelial cells (HUVECs). The anti-angiogenic activity of SHED-Exos was assessed via a tube formation assay of endothelial cells and angiogenesis-related factors were analyzed by western blotting. In vivo, we used the chick chorioallantoic membrane (CAM) assay and an oral squamous cell carcinoma (OSCC) xenograft transplantation model with nude mice that received multi-point injections at three-day intervals to evaluate the effects on angiogenesis. Furthermore, the sequencing of microRNAs (miRNAs) in SHED-Exos was performed to investigate the underlying anti-angiogenic mechanism.

**Results:**

The results showed that SHED-Exos inhibit cell proliferation and migration and induce apoptosis in HUVECs. SHED-Exos suppress the tube-like structure formation of HUVECs in vitro. SHED-Exos downregulate several angiogenesis-related factors, including VEGFA, MMP-9 and ANGPT1. In vivo, the chick CAM assay verified that treatment with SHED-Exos inhibits micro-vascular formation, and importantly, significantly reduces the micro-vascular formation of tumors generated from xenografted OSCC cells, which was associated with the inhibition of tumor growth in vivo. Mechanistically, our data suggested that SHED-Exos are enriched with miR-100-5p and miR-1246 and are transferred to endothelial cells, which results in decreased tube formation via the down-regulation of VEGFA expression.

**Conclusions:**

These results demonstrate that SHED-Exos inhibit angiogenesis in vitro and in vivo, which suggests that SHED-Exos could potentially serve as a novel and effective therapeutic approach for anti-angiogenic treatment.

**Supplementary Information:**

The online version contains supplementary material available at 10.1186/s13287-022-02764-9.

## Background

Angiogenesis, the process of developing new capillaries from preexisting blood vessels, is recognized as a pivotal process both in physiological and in pathological conditions with a sufficient supply of oxygen and nutrition [1–3]. Tumor angiogenesis is also particularly critical for tumor growth. Without that process, the tumor volume cannot exceed 3 mm^3^ [4]. Based on this, anti-angiogenic therapy has been shown to be a promising therapeutic target in anti-cancer treatment, for example, bevacizumab, an angiogenesis inhibitor, was approved and used for treatment of several cancers [5].

Mesenchymal stem cells (MSCs) are multipotent stem cells in various tissues (e.g., bone marrow, embryonic tissue and dental tissue) that have been studied extensively for cancer treatment [6–8]. It has been reported that the therapeutic effects of MSCs-based therapies are mainly due to the paracrine secretion of bioactive factors and exosomes have been recognized to be important paracrine mediators for intercellular communication [9–12]. Exosomes are membrane-bound vesicles approximately 30–150 nm in diameter that can be secreted by many types of cells. These small vesicles originate from early endosomes and as they mature into late endosomes, the number of intraluminal vesicles increases, and they are then released upon the fusion of endosomes with the cell surface [13]. Exosomes carry internal bioactive molecules shuttled by donor cells, including microRNAs (miRNAs), mRNAs, proteins and lipids that can regulate gene expression in the recipient cells, such as endothelial cells, to affect their growth, migration and/or angiogenesis [14–16]. Pakravan et al. reported that bone marrow MSCs-derived exosomes could suppress the secretion and expression of VEGF in breast cancer derived cells, and furthermore, could inhibit in vitro angiogenesis of endothelial cells [17]. However, other studies reported MSCs-derived exosomes promote angiogenesis. For instance, Xue et al., reported that human adipose MSCs-derived exosomes were shown to stimulate the angiogenesis of human brain microvascular endothelial cells [18]. Therefore, it will be important to further test the different roles of exosomes derived from different types of MSCs in the regulation of angiogenesis.

Stem cells of deciduous exfoliated teeth (SHED) have unique properties compared with other MSCs. Primarily, obtaining SHED cells represents a noninvasive and accessible method of harvesting stem cells, because deciduous teeth are naturally discarded after exfoliation. These cells can be easily obtained after exfoliation with patient's safety assured and with fewer ethical concerns [19]. Furthermore, SHED cells, considered to be one type of immature MSCs from human dental tissue, exhibits a stronger proliferative ability and capability for osteogenic and odontogenic differentiation and possesses a more prominent and versatile differentiation ability than adult dental pulp stem cells (DPSC) [20–22]. In addition, SHED cells, present before birth and maintained before the eruption of permanent teeth, is characterized by maintenance of an active niche rich in stem cells, which are not yet heavily affected by the cumulative effects of genetic and/or environmental factors [23]. Although the MSCs-exosomes field has developed rapidly, the biological characteristics and functions of SHED-derived exosomes (SHED-Exos), especially the role of SHED-Exos in angiogenesis, remains poorly characterized. Therefore, the purpose of this study was to investigate what role SHED-Exos play in angiogenesis and to explore the underlying molecular mechanism.

## Materials and methods

### Cell isolation and cultivation

Subjects with deciduous incisors with at least more than one-half of the physiological root who had been scheduled for extraction were enrolled in our study. These teeth were collected from six donors aged 6 to 8 years old. SHED cell isolation and culture were performed as previously described [24]. SHED cells were cultured in Dulbecco’s Modified Eagle Medium (DMEM, Cat. 11,995,500, Thermo Fisher Scientific) with 1 g/L D-Glucose supplemented with 10% fetal bovine serum (FBS, Cat. 16,140,071, Thermo Fisher Scientific) and 1% penicillin–streptomycin (Cat. 10,378,016, Thermo Fisher Scientific). SHED cells at passages 3 to 6 were used for these experiments. Human umbilical vein endothelial cells (HUVECs) were purchased from AllCells (Cat. H-001F, AllCells) and were cultured in DMEM (Cat. SH30021.01, Hyclone) with 4.5 g/L D-Glucose supplemented with 10% FBS. Cells were cultured at 37 °C under 5% CO_2_.

### Isolation and characterization of SHED-Exos

SHED cells were cultured until approximately 80% confluent and were washed three times with phosphate-buffered saline (PBS, Cat. 10,010,049, Thermo Fisher Scientific) and then incubated in DMEM with 10% exosomes-deprived FBS obtained by centrifugation at 100,000 × g at 4 °C for 10 h. After incubation for 48 h, conditioned medium (CM) was collected and centrifuged at 3,000 × g at 4 °C for 10 min followed by filtering through 0.2 μm filters [25]. An exosome concentration solution (ECS) kit (Cat. UR52121, Umibio) was used to isolate and purify exosomes following the manufacturer’s protocol. Briefly, ECS was added into the prepared CM and was then stored at 4 °C for 2 h after intensive mixing. Following centrifugation at 10,000 × g for 60 min at 4 °C, the precipitates were resuspended in PBS and purified with a purification filter at 3,000 × g for 10 min at 4 °C and then stored at − 80 °C.

The concentration of exosomes was calculated using a bicinchoninic acid (BCA) protein quantitation kit (Cat. PC0020, Solarbio). The characteristics of exosomes derived from SHED cells were further identified. Firstly, the morphology of exosomes was observed using a transmission electron microscope (TEM, G2 spititi FEI, Tecnai). Nanoparticle tracking analysis (NTA) using ZetaView Particle Metrix (ZetaView PMX 110, Particle Metrix) was then used to analyze the particle size distribution of exosomes and western blotting was performed to detect the exosome-specific markers CD63 (Cat. Ab216130, Abcam) and TSG101 (Cat. Ab125011, Abcam).

### Labeling of exosomes and uptake by HUVECs

Exosomes derived from SHED cells were labeled with PHK67 fluorescent dye (Cat. UR21028, Umibio). In brief, 5 μl PKH67 dye was added to 30 μg exosomes in a total of 50 μl diluent C provided in the kit and incubated at room temperature for 10 min. Exosomes were re-isolated and re-purified according to the method of exosome isolation. HUVECs were incubated with the labeled exosomes at 37 °C for 24 h and images of ingested exosomes were obtained using an inverted fluorescence microscope (Olympus, Japan).

### Cell counting Kit-8 assay (CCK8 assay)

CCK8 assays were performed using a Cell Counting Kit-8 (Cat. 35,532,286, Dojindo) to evaluate cell viability. Briefly, a total of 5 × 10^3^ HUVECs per well were seeded into 96-well plates and incubated with exosomes (30 μg/ml) or PBS (control). Five wells were designed for each group. At specific time points, 10 μl CCK8 working solution was added into each well. After incubation for 1.5 h at 37 ℃, the optical absorbance was quantified at 450 nm wavelength.

### Ki67 immunofluorescent staining

Immunofluorescence staining of Ki67 was performed to examine cell proliferation ability. Briefly, cells were seeded on a microscope cover glass in 24-well plates, which were permeabilized using 0.5% Triton X-100 (Cat. 9036–19-5, Sigma-Aldrich). After washing three times with PBS, 10% goat serum was used for blocking. The cells were then incubated with Ki67 primary antibody (Cat. Ab15580, Abcam) overnight at 4° C. On the second day, the cells were washed three times with PBS and incubated with secondary antibodies in the dark at room temperature for 1 h. Cell nuclei were stained using DAPI (Cat. Ab104139, Abcam) for 5 min, and stained images were captured using a BX53-DP80 immunofluorescence microscope (Olympus, Japan). The cell proliferation rate was defined as follows: the number of proliferating cells / total number of cells × 100%.

### Motility and migration assays

Scratch wound healing assays were used to evaluate cell motility. Briefly, 3 × 10^4^ HUVECs were seeded in six-well plates. Following growth to 100% confluence, cells were subjected to single vertical scratches using a 200 μl pipette tip. After washing three times with PBS, SHED-Exos (30 μg/ml) or PBS (control) were added, and images were recorded at 0 and 24 h after scratching using an optical microscope (Leica, Germany). The rate of wound closure was calculated as follows: (mean wound width—mean remaining width) / mean wound width × 100%.

Transwell migration assays were used to determine the effects of SHED-Exos on the ability to recruit HUVECs. In brief, 1 × 10^4^ HUVECs were seeded into the upper chamber of 24-well transwell plates containing an 8 μm pore size membrane in each well (Cat. 3422, Corning). The upper chamber contained 100-μl serum-free DMEM with SHED-Exos (30 μg/ml) or PBS (control) and 500 μl/well medium with 10% exosomes-deprived FBS obtained by centrifugation at 100,000 × g at 4 °C for 10 h added into the lower chamber. After incubation for 24 h, the cells that had migrated into the bottom of the chamber were fixed with 4% paraformaldehyde for 10 min and then were stained with 0.5% crystal violet for 7 min. After removing the cells at the upper surface of the membrane, the stained cells were recorded and calculated.

### Flow cytometry (FACS)

The surface markers of SHED cells were examined using a flow cytometer (Calibur, BD Biosciences). 1 × 10^6^ SHED cells were incubated with following fluorochrome-conjugated antibodies: anti-CD44-APC (Cat. MA1-10,226, Invitrogen), anti-CD105-PE (Cat. 12–1057-42, Invitrogen), anti-CD90-PE (Cat. 12–0909-42, Invitrogen), anti-CD45-FITC (Cat. 14–9457-95, Invitrogen), anti-CD19-APC (Cat. 17–0199-42, Invitrogen) and anti-CD14-PerCP (Cat. 450,149–42, Invitrogen) for 1 h at 4℃ in the dark and were then analyzed after washing in PBS.

The percentage of cell apoptosis was determined by FACS. In brief, 3 × 10^4^ HUVECs per well were seeded into six-well plates. Following incubation with SHED-Exos (30 μg/ml) or PBS (control) for 48 h, cells in the supernatant were collected, centrifuged and incubated with 500 μl binding buffer containing 10 μl PI and 5 μl Annexin V-FITC (Cat. 556,419, BD Biosciences) for 10 min at room temperature in the dark and were then analyzed by FACS.

### Quantitative real-time PCR (qRT-PCR)

Total RNAs were extracted from cells and from SHED-Exos using the Trizon reagent (Cat. 50,175,111, Thermo Fisher Scientific). RNA concentrations were measured using a Nanodrop spectrophotometer (Thermo Fisher Scientific, Shanghai, China), and each RNA was then reverse transcribed to complementary DNA (cDNA) using a superscript III first strand kit (Cat. 18,080–05, Invitrogen). qRT-PCR reactions were performed with the SYBR Green qPCR Mix (Cat. 9211, Biosharp) in a total volume of 10 μl using a LightCyclerR 96 (Roche Diagnostics, Rotkreuz, Switzerland) according to the manufacturer’s instructions, with the following parameters: 95 °C for 30 s, 40 cycles at 9 °C for 5 s, at 60 °C for 20 s and ended with an elongation step for 15 s at 72 °C. GAPDH was used as an internal control. The PCR primers used are shown in the Supplementary Table.

Similarly, following cDNA synthesis using an All-in-one™ miRNA first-stand cDNA synthesis kit (Cat. AMRT-0020, GeneCopoeia), miRNA qRT-PCR reactions were performed using the All-in-one™ miRNA qPCR kit (Cat. AMRP-1200, GeneCopoeia) in a total volume of 10 μl. U6B small nuclear RNA (RNU6B) was chosen to normalize the expression of miRNAs [26]. The relative expression levels were determined using the 2^−ΔΔCt^ method. All primers used for miRNA qRT-PCR were purchased from GeneCopoeia, Guangzhou, China.

### Western blot analysis

Firstly, cells were washed with ice-cold PBS and total proteins were lysed with radioimmunoprecipitation assay (RIPA, Cat. 89,900, Thermo Fisher Scientific) buffer containing 1% phosphatase inhibitor cocktail and 1% phenylmethylsulfonyl fluoride (PMSF, Cat. 36,978, Thermo Fisher Scientific) for 30 min at 4 °C. Protein concentrations were calculated using a BCA protein quantitation kit (Cat. PC0020, Solarbio). Equal amounts of protein were loaded and separated on 12% SDS-PAGE gels and then were transferred to polyvinylidene fluoride (PVDF) membranes (Cat. 88,518, Thermo Fisher Scientific) according to standard protocols. The membranes were blocked with 5% non-fat milk powder dissolved in Tris-buffered saline containing 0.05% Tween-20 (TBST, Cat. T1081, Solarbio) and then were incubated with the primary antibodies overnight at 4 °C. After washing with TBST buffer, the membranes were incubated with the secondary antibodies for 2 h. Signal detection was visualized by enhanced chemiluminescence reagents (Cat. SW2050, Solarbio), and immunoreactive bands were quantified using Image J (National Institutes of Health, USA) with GAPDH as the reference control.

The primary and secondary antibodies used were as follows: VEGFA (Cat. Ab1316, Abcam), MMP-9 (Cat. Ab228402, Abcam), ANGPT1 (Cat. EPR2888N, Abcam), PARP (Cat. 13,371–1-AP, Proteintech), total Caspase 3 (Cat. 9662, CST), cleaved-Caspase 3 (Cat. 9664, CST), total Caspase 9 (Cat. Ab202068, Abcam), GAPDH (Cat. Ab181602, Abcam), HRP anti-rabbit (Cat. 7074, CST) and HRP anti-mouse (Cat. 7076, CST).

### Tube formation assay

Tube formation assays were conducted according to the manufacturer’s instructions. In brief, 50 μl dissolved Matrigel (Cat. 354,230, Corning) were added to pre-cooled 96-well plates and were incubated at 37 ℃ for 1 h. Three × 10^4^ HUVECs were seeded into each Matrigel-coated well. The network structures were captured using phase contrast microscopy (Leica, Germany) and were quantified using Image J software.

The tube formation ability of HUVECs was compared between the SHED-Exos treated group (30 μg/ml) and the control group. The tube formation ability was compared among four groups: PBS, CW, CM + GW4869, CM + GW4869 + Exos. SHED-Exos were pre-incubated with culture medium supplemented with 10 μm/L GW4869 (Cat. UR21021, Umibio) according to the manufacturer’s instructions. After 48 h, the CM (CM + GW4869 group) was collected for the tube formation assay.

### Chick chorioallantoic membrane (CAM) assay

Chick CAM membrane assays were used to evaluate the anti-angiogenesis effects of SHED-Exos in vivo. Fertilized chicken eggs were disinfected using 75% alcohol and were then incubated at 37.8℃. After eight days, small openings were made at the top of the air chamber of the shell. Subsequently, a small hole was made in middle of each CAM using tweezers and SHED-Exos or PBS was injected into the hole. The openings were covered with sterile tape and were then returned to the incubator. After incubation for 48 h, the CAMs were cut out and the pictures were taken using a stereo microscope (Olympus, Japan). The number of micro-vessels around the injection area were calculated. Five samples were designed in each group.

### Animal models

A total of 25 four-week-old female BALB/C nude mice (Cat. 403, Charles River, Beijing, China) were used. Eight × 10^4^ Cal27 cells suspended in 200 μl PBS were subcutaneously injected into the left flank of each nude mouse to establish the xenogeneic oral squamous cell carcinoma (OSSC) transplantation model. The tumor growth was monitored by measuring the length and width using an electronic vernier caliper every three days, and the tumor volume was calculated as 0.5 × length × width^2^ [27]. When the tumors reached 20 mm^3^, mice were randomly divided into a SHED-Exos treated group (30 μg exosomes suspended in 50 μl PBS) and a PBS (control) group. We performed multi-point intratumoral injections near the base of the tumor four times at three day intervals using a micro-syringe with a 30-gauge needle (Cat. 1700, Hamilton). Tumor tissues were harvested three days after the last injection and the tumors were isolated for photography. Immunohistochemical staining for CD31 (Cat. Ab28364, Abcam) was performed to analyze the pathological characteristics of the tumor.

### Sequencing analysis of exosomal miRNAs

To further characterize the miRNA cargoes of SHED-Exos, miRNAs were sequenced in three independent experiments by LC-Bio Technology (Hangzhou, China). The top 15 highly expressed miRNAs were listed according to their expression level, which was defined as the normalized data of the number of reads for each isoform at the 5' or 3' end of the corresponding miRNAs.

### Transfection of specific mimics and inhibitor into HUVECs

To explore the functions of miRNAs in anti-angiogenic action, HUVECs seeded into six-well plates were treated with specific miRNA mimics or inhibitor using Lipofectamine 3000 (Cat. L000001, Thermo Fisher Scientific). Briefly, 12.5 μl of each specific miRNA mimics or inhibitor diluted in 125 μl Opti-MEM™ medium (Cat. 31,985,088, Thermo Fisher Scientific) was allowed to stand for 5 min, after which 7.5 ul Lipofectamine 3000 in 125 μl Opti-MEM™ medium were incubated for 5 min. Following mixing and standing for 10 min at room temperature, all reagents mentioned above were added into HUVECs prepared in six-well plates with around 80% confluency. Following incubation for 24 h, cells were changed to fresh medium for another 24 h, after which the tube formation assay and specific genes related to angiogenesis were assessed.

### Dual luciferase reporter assay

The dual luciferase reporter assay was performed as previously reported [28, 29] and was used to analyze whether miR-100-5p directly targets mTOR to control its expression. Wild-type mTOR (mTOR-WT) 3′-UTR containing the miR-100-5p potential binding site (5' TACGGGT 3') was inserted into the pmirGLO luciferase vector (Promega Corporation). A cDNA fragment of the mutant sequence of mTOR (mTOR-MuT) 3′-UTR with a target region (5' GCATTTG 3') was also inserted into the pmirGLO vector. mTOR-WT or mTOR-MuT with miR-100-5p mimics or mimics NC were cotransfected into 293 T cells using Lipofectamine 3000 and incubated for 36 h. The relative luciferase activity was detected using a Dual-Luciferase® Reporter assay system (Promega Corporation).

### Statistical analysis

All experiments were conducted in triplicate. The results are presented as means ± standard deviation (SD). Comparisons between experimental groups with control groups were determined by Student’s t test using SPSS software (SPSS, IBM Corp. Version 22.0. Armonk, New York, USA). Double factor analysis of variance (ANOVA) was performed when comparing two groups with more independent variables. P < 0.05 was considered significant.

## Results

### SHED-Exos are efficiently taken up by endothelial cells

**Fig. 1:**
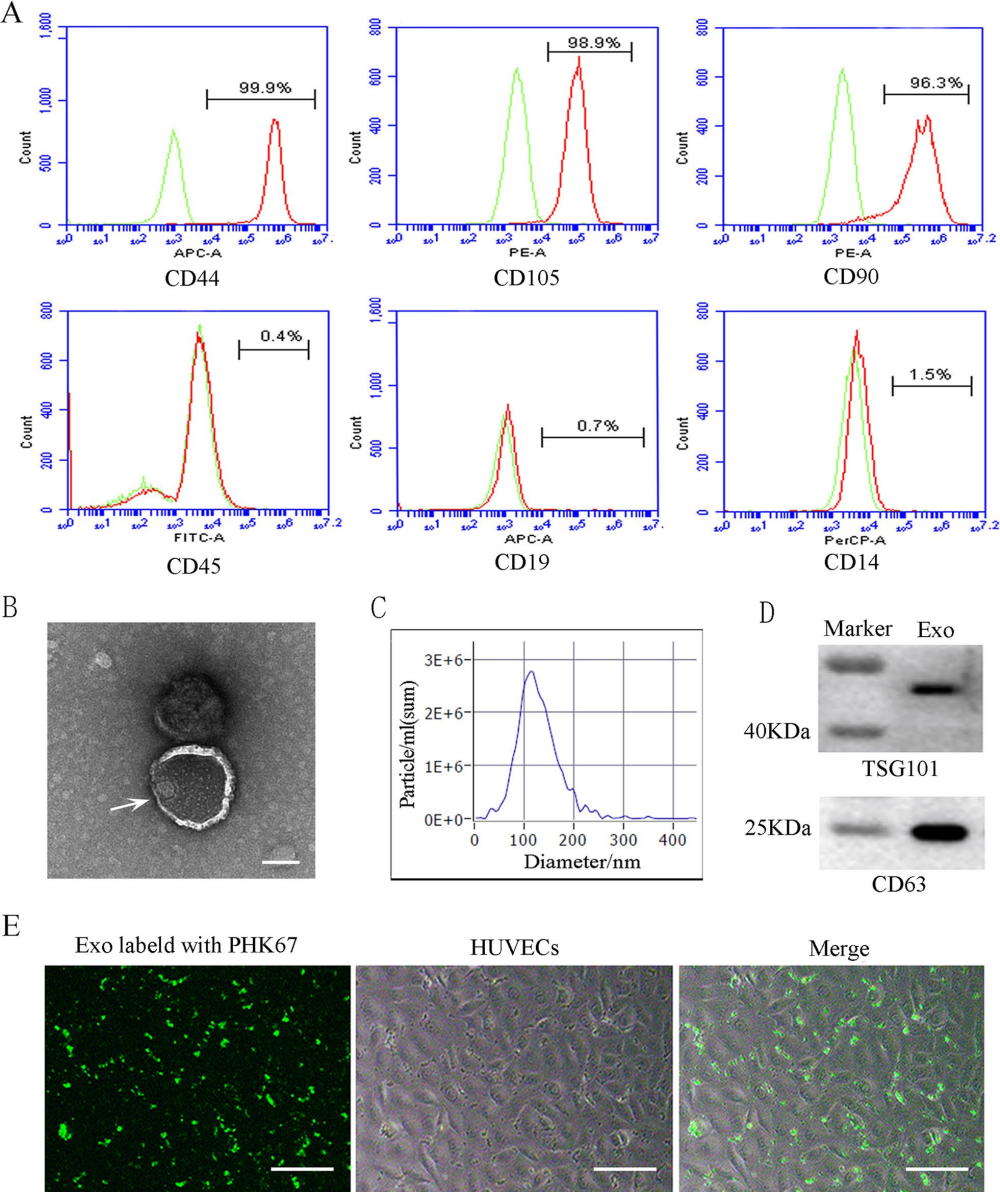
Characterization and uptake of SHED-Exos. **A** Surface markers of SHED cells were analyzed by flow cytometry (FACS) and were positive for mesenchymal markers (CD44, CD105 and CD90) and negative for endothelial markers (CD45, CD19 and CD14). **B** The morphology of exosomes (indicated by arrows) was observed using a transmission electron microscope (TEM). Scale bar = 100 nm. **C** Particle size distribution of SHED-Exos assessed by nanoparticle tracking analysis (NTA). **D** Expression of exosome-specific CD63 and TSG101 validated using western blotting. **E** Efficient uptake of PKH67-labeled exosomes by HUVECs was detected at 24 h. Scale bars = 100 μm

In order to obtain SHED-Exos, we first verified that the isolated SHED cells were MSCs by analyzing specific MSCs surface markers using flow cytometry. Flow cytometric assays showed that SHED cells expressed high levels of the MSCs markers CD44, CD105 and CD90, but did not express any endothelial cell marker, i.e., CD45, CD19 and CD14 (Fig. 1A). Next, we set out to identify the characteristics of SHED-Exos. Specifically, SHED-Exos were analyzed using a transmission electron microscope (TEM), which revealed the typical cup shape of exosomes (Fig. 1B). The diameter of those exosomes was assessed by nanoparticle tracking analysis (NTA) and was shown to range from 30 to 150 nm (Fig. 1C). Additionally, expression of the exosome-specific markers CD63 and TSG101 was detected by western blotting of SHED-Exos (Fig. 1D). Taken together, these results validated that the extracellular vesicles isolated from SHED cells are exosomes. Subsequently, to test the potential biological functions of SHED-Exos, the exosomes were labeled with PHK67 dye, and the PHK67-labeled exosomes were added to HUVECs. As shown in Fig. 1E, the PHK67-labeled SHED-Exos were efficiently taken up by endothelial cells after incubation for 24 h, indicating that the SHED-Exos potentially produced effects on endothelial cells.

### SHED-Exos significantly inhibit the growth and induce the apoptosis of endothelial cells in vitro

**Fig. 2:**
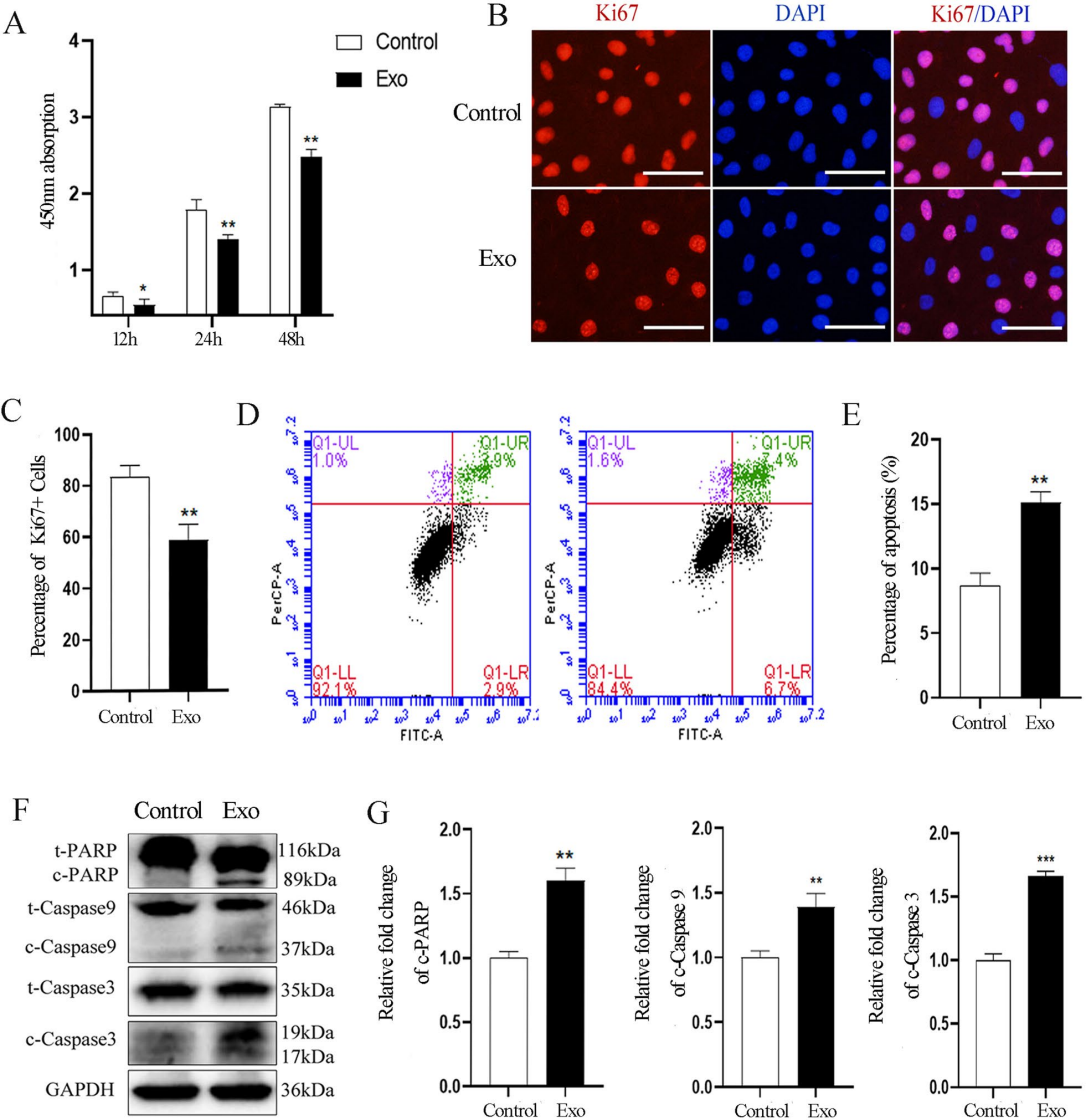
SHED-Exos inhibit growth and induce apoptosis of endothelial cells. **A** Viability of endothelial cells analyzed using the CCK-8 assay at different time points. **B** Immunofluorescence staining of Ki67 to test the proliferation viability of endothelial cells. Scale bars = 500 μm.** C** Quantification of Ki67-positive cell percentage (%) in **B**. **D** HUVECs were incubated with SHED-Exos or PBS (control) for 48 h and analyzed for apoptosis by FACS. **E** Quantification of apoptotic cell percentage (%) in **D**. **F** Western blotting analysis of total and cleaved proteins of PARP, Caspase 9 and Caspase 3 in endothelial cells incubated with exosomes or PBS (control). GAPDH is a housekeeping gene used as a loading control. **G** Quantification of the relative levels of c-PARP, c-Caspase 9 and c-Caspase 3 in **F**, as relative change to the respective control cells, after each cleaved protein was normalized to the corresponding total protein band. All experiments were performed three times, and error bars represent means ± SD; P values are indicated with “*”, * indicates P < 0.05, ** indicates P < 0.01, *** indicates P < 0.001 when comparing SHED-Exos-treated cells with the control group in **C**, **E** and **G** by Student’s t test

To determine whether SHED-Exos affect the growth of endothelial cells, we used CCK8 assays to evaluate cell viability after incubation with SHED-Exos (30 μg/ml). We found that treatment with SHED-Exos significantly inhibited the growth of endothelial cells after incubation for 12 h, and the inhibitory effect became more significant at 24 h and 48 h (Fig. 2A). In order to further evaluate whether SHED-Exos have an inhibitory effect on cell proliferation, we performed Ki67 immunofluorescent staining, which showed that the positive expression rate of Ki67 was 59.2 ± 4.2% in the SHED-Exos treated group, which was significantly lower than that of the control group (82.1 ± 2.9%) (Fig. 2B, C). Taken together, these data suggested that SHED-Exos significantly inhibit endothelial cell growth in vitro.

The inhibition of endothelial cell growth might also be due to increased cell death elicited by SHED-Exos, and therefore, we next investigated whether they induced the apoptosis of endothelial cells, which is recognized as a crucial type of cell death. First, flow cytometric analysis using annexin V-FITC/PI double-staining showed that incubation of HUVECs with SHED-Exos dramatically increased the percentage of apoptotic cells. Compared with the control group (8.1 ± 2.1%), the apoptosis rate of the SHED-Exos treated group increased to 15.6 ± 1.9% at 48 h (Fig. 2D, E). Since the activation of caspase pathways is usually involved in the apoptosis induction, western blotting was performed to confirm the involvement of caspase activity in the SHED-Exos induced apoptosis. The levels of cleaved Caspase 9 (c-Caspase 9), cleaved Caspase 3 (c-Caspase 3) and the protein cleaved PARP (c-PARP) significantly increased after treatment with SHED-Exos (Fig. 2F, G). These findings suggest that SHED-Exos significantly induce the apoptosis of endothelial cells in vitro.

Taken together, these data demonstrate that SHED-Exos can inhibit the growth of endothelial cells both by suppressing their proliferation and by promoting their apoptosis.

### SHED-Exos significantly suppress the migration of endothelial cells in vitro

**Fig. 3:**
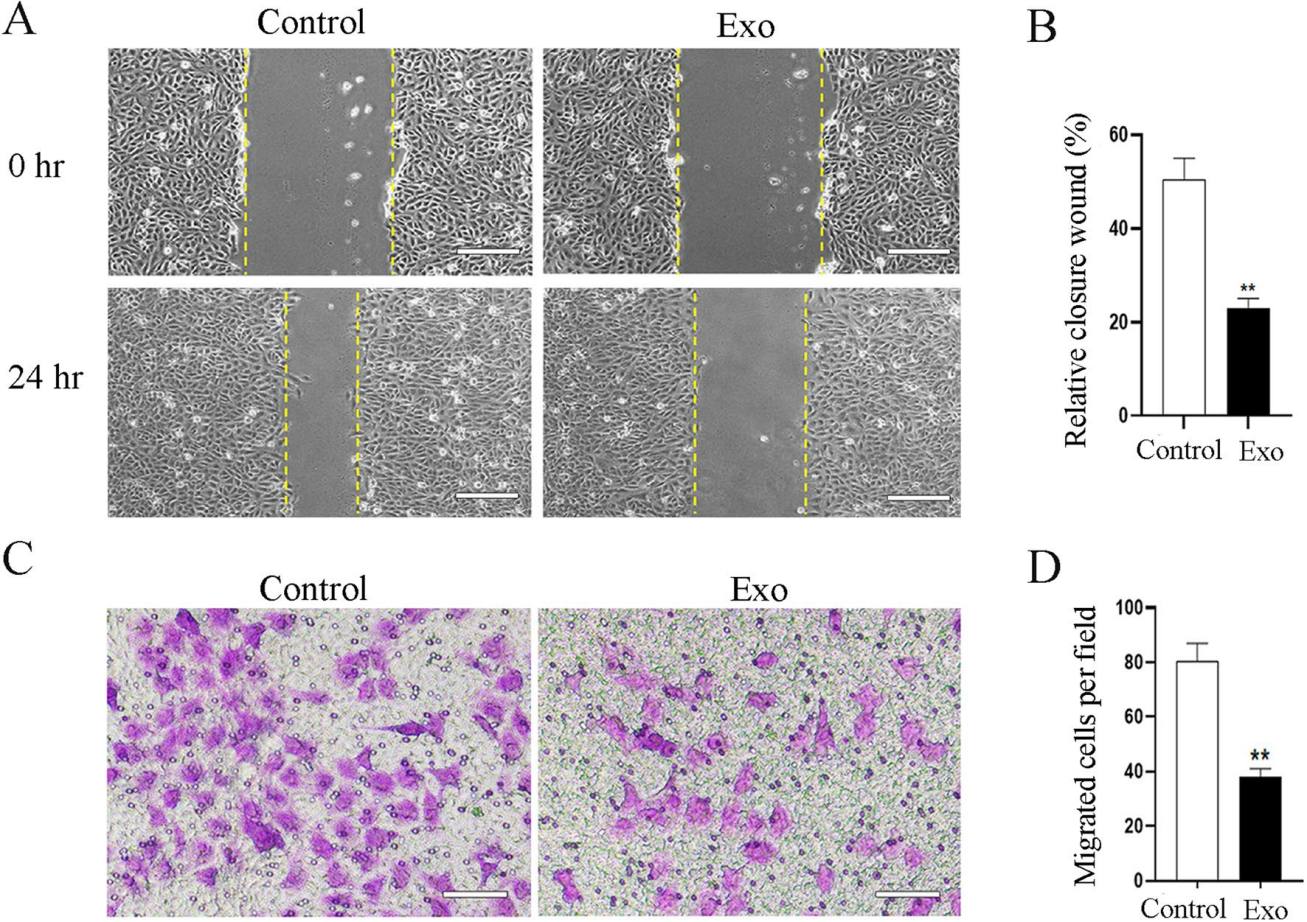
SHED-Exos suppress the migration of endothelial cells. **A** Wound healing assay demonstrating the significantly decreased closure of the scratch area in the SHED-Exos-treated group compared with the control group. Scale bars = 200 μm. **B** Quantification of the rate of scratch closure (%) in **A**. **C** Transwell migration assays revealing that a significantly lower number of HUVECs migrated through membranes with SHED-Exos than with PBS. Scale bars = 200 μm. **D** Quantification of the number of migrated cells per field in **C**. P values are indicated with *, * Indicates P < 0.05, ** indicates P < 0.01, *** indicates P < 0.001, when comparing SHED-Exos-treated cells with the controls in **B** and **D** by Student’s t test

It has been well-established that the migration and invasion of adjacent endothelial cells are important actions for the growth of new micro-vascular networks from preexisting blood vessels during vascular development. Therefore, we tested whether SHED-Exos could inhibit the migration behavior of HUVECs in vitro. To this end, we used a scratch wound healing assay, which demonstrated that endothelial cells treated with 30 μg/ml SHED-Exos had a significantly lower rate of wound closure than PBS treated cells (Fig. 3A, B). To further confirm this observation, we carried out Transwell migration assays, which revealed that a significantly lower number of endothelial cells migrated to the bottom compartment in response to treatment with SHED-Exos (Fig. 3C, D, Additional file 1: Fig. S1). In sum, these results indicate that SHED-Exos can suppress the migration and invasion of endothelial cells in vitro.

### SHED-Exos inhibit tube formation of endothelial cells in vitro

**Fig. 4:**
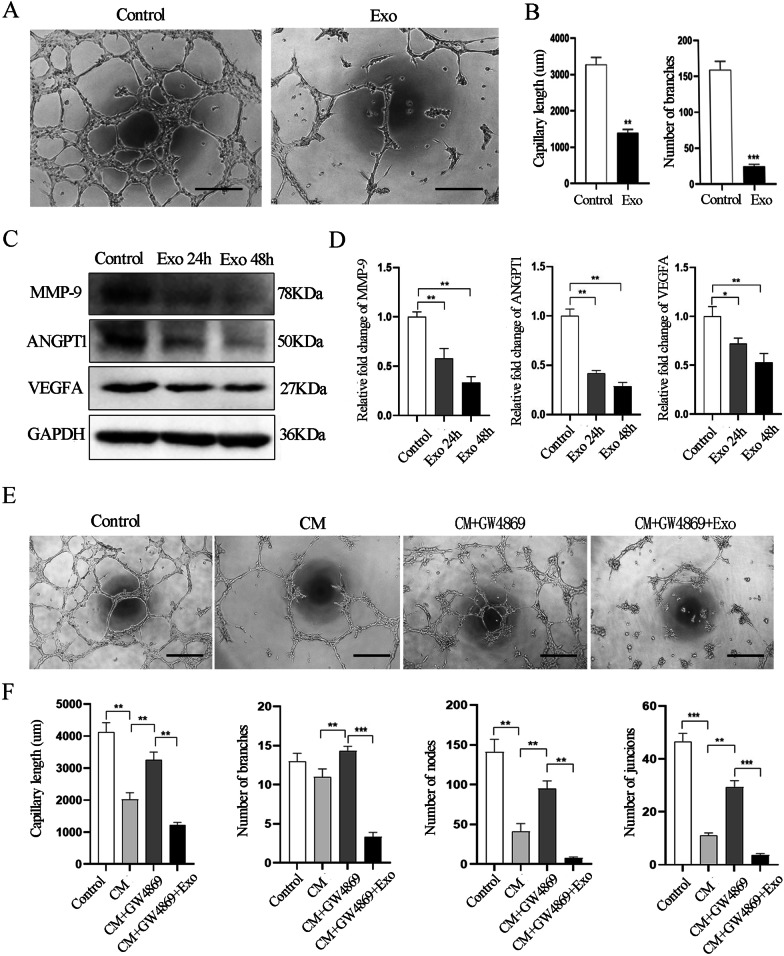
SHED-Exos inhibit the tube formation of endothelial cells in vivo. **A** Matrigel tube formation assay showing the inhibitory effect of SHED-Exos on capillary-like structures formed by HUVECs. Scale bars = 300 μm. **B** Quantification of tube length and number of tube branches in the network structures of HUVECs in **A**. **C** Western blot analysis showing the protein expression levels of VEGFA, MMP-9 and ANGPT1. **D** Quantification of the relative levels of VEGFA, MMP-9 and ANGPT1 in **C** as relative fold change to the respective control cells. **E** Matrigel tube formation assay of the PBS group, the CM group, the CM + GW4869 group and the CM + GW4869 + Exo group. Scale bars = 300 μm. **F** Quantification of tube length and number of branches in the network structures of HUVECs in **E**. Means and standard deviations are shown in each group in **B**, **E**, **G** and **I**. *P < 0.05, **P < 0.01, ***P < 0.001

Based on the observations of the inhibitory effect of SHED-Exos on the proliferation and migration of endothelial cells in vitro, we speculated that SHED-Exos might directly affect the angiogenesis ability of endothelial cells. To test that hypothesis, we performed tube formation assays in vitro. Shorter tubes and fewer branches, nodes and junctions of the network structures of endothelial cells were observed in the SHED-Exos treated group compared to the control group (Fig. 4A). Quantification of those results (Fig. 4B and Additional file 1: Fig. S2) indicated that difference was statistically significant (P < 0.05). Next, we evaluated whether the inhibitory effect on tube formation was through the negative regulation of angiogenesis-related factors. We incubated HUVECs with SHED-Exos for 24 h and 48 h and examined their expression of angiogenesis-related proteins. Western blot results showed that the protein expression level of VEGFA, which is known as a crucial angiogenesis molecule, was significantly reduced at 24 h and 48 h compared with PBS treated cells. Concurrently, other proangiogenic factors, including MMP-9 and ANGPT1, were also downregulated by treatment with SHED-Exos (Fig. 4C, D). Overall, these results suggest that SHED-Exos inhibit the tube formation of endothelial cells in vitro associated with the downregulation of proangiogenic factors.

To further confirm the above findings, we performed Matrigel tube formation assays by incubating HUVECs with the CM collected from SHED cells cultured for 48 h and with DMEM containing 10% exosomes-depleted FBS as a positive control. Our results showed that there was much less tube formation (Fig. 4A) in HUVECs treated with SHED derived CM, including decreases in tube length, branches, nodes and junctions in the network structures of HUVECs (Fig. 4B, Additional file 1: Fig. S2). To further validate that the inhibitory effect of CM was due to the exosomes, we pretreated SHED cells with GW4869, a well-known inhibitor of exosome secretion before collecting the CM [30]. We found that the inhibitory effect of the CM on tube formation was mostly abrogated by pretreatment with GW4869 (Fig. 4E, F). Notably, when SHED-Exos were added to the CM with pretreatment of GW4869 (CM + GW4869 group), the addition of SHED-Exos suppressed the tube formation of HUVECs again (Fig. 4E) with corresponding reductions in tube length, number of branches, nodes and junctions in the network structures of HUVECs (Fig. 4E, F). Taken together, these data suggest that SHED cells can secrete exosomes into culture medium which inhibit the tube formation of endothelial cells, as a paracrine mediator in vitro.

### SHED-Exos suppress in vivo micro-vascular formation, which potentially plays a crucial role in anti-tumor function

**Fig. 5:**
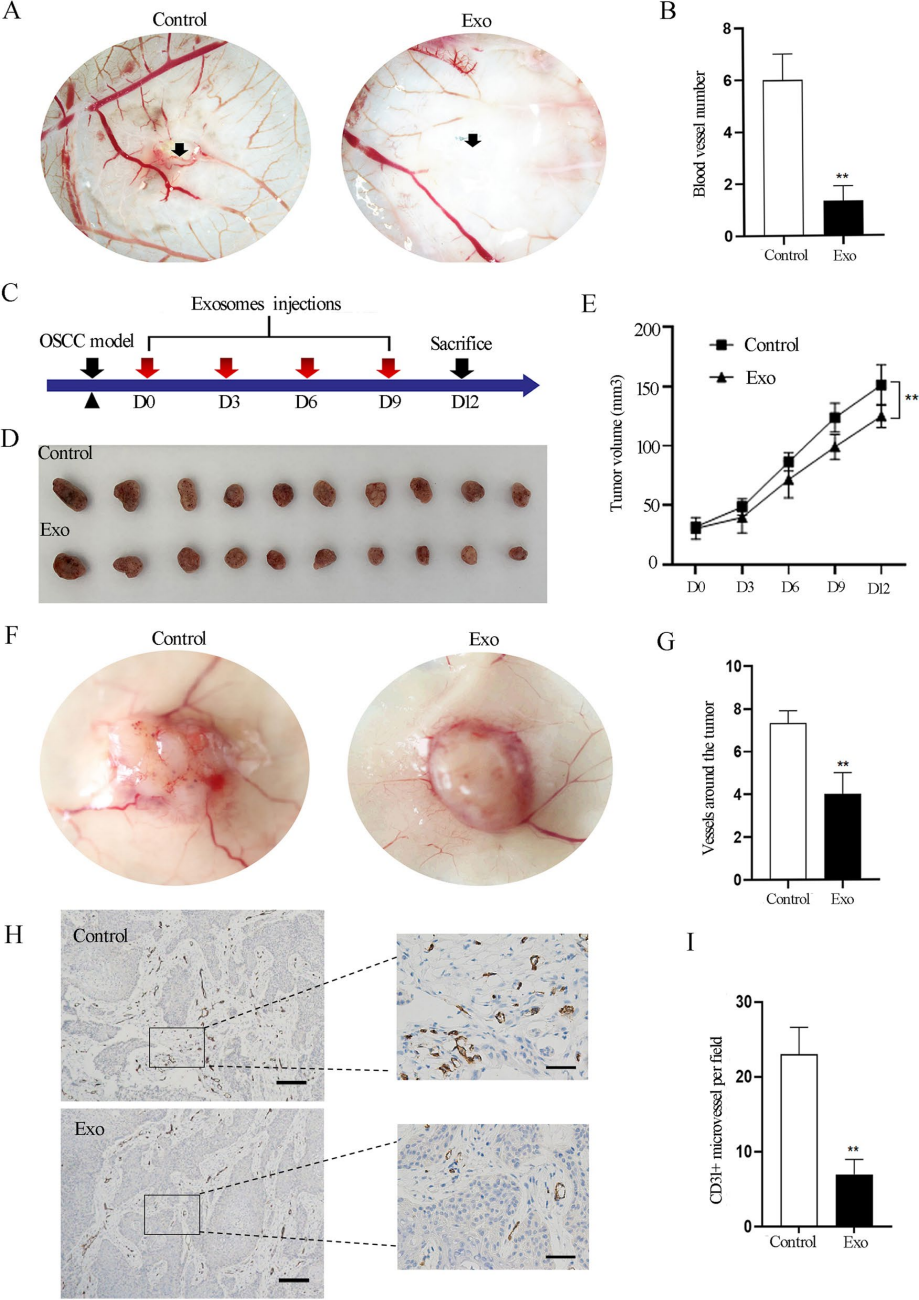
SHED-Exos suppress micro-vascular formation in vivo. **A** CAM assays evaluating the anti-angiogenesis ability of SHED-Exos; arrows indicate the injection area. **B** Quantification of the number of micro-vessels in **A**. **C** Schematic diagram of the experimental plan. **D** Representative images of OSSC tumors harvested from mice three days after the last intra-tumoral injection of SHED-Exos or PBS (control). **E** Tumor volumes at different time points. **F** Representative images of micro-vessels around the base of the harvested tumors. **G** Quantification of the number of vessels in **D**. **H** Immunohistochemical staining for CD31 after treatment with SHED-Exos or PBS (scale bars = 200 μm). Images on the right show higher magnifications of areas inside the square boxes on the left (scale bars = 50 μm). **I** Quantification of the number of CD31 positive cells in the magnified images shown in **H**. Means and standard deviations are shown in each group in **B**, **E**, **G** and **I**. *P < 0.05, **P < 0.01, ***P < 0.001

Next, to test whether SHED-Exos also have a suppressive effect on micro-vascular formation in vivo, we performed CAM assays, a classic in vivo model to study the angiogenesis of endothelial cells [31]. Fertilized chicken eggs were incubated at 37.8℃ for eight days and then the SHED-Exos or PBS (control) were injected via small holes in the middle of each CAM. After incubation for 48 h, the CAMs were cut out and the number of micro-vessels around the injection area was calculated. The results showed that the number of formed micro-vascular was significantly decreased in the SHED-Exos-treated group (Fig. 5A, B), demonstrating that SHED-Exos significantly suppress micro-vascular formation in vivo*.*

Based on the observation of the inhibitory effect on the tube formation of endothelial cells both in vitro and on micro-vascular formation in vivo, we speculated that SHED-Exos may play an anti-tumor function through the inhibition of angiogenesis of tumor tissues in vivo*.* To test this possibility, we established a xenogeneic transplantation model of OSCC, which is one of the most prevalent malignant tumors of the oral cavity. We performed multi-point intratumoral injections of SHED-Exos suspended in PBS as shown in Fig. 5C, after which tumor growth was monitored by measuring the length and width at 3 day intervals to calculate the tumor volume. We found that SHED-Exos treated tumors maintained volumes of 30.70 ± 9.25 mm^3^ to 40.14 ± 13.20 mm^3^, 71.73 ± 15.40 mm^3^, 99.17 ± 10.57 mm^3^ and 125.04 ± 9.77 mm^3^ at days 0, 3, 6, 9 and 12, respectively. On the other hand, the untreated control tumors grew steadily from 32.13 ± 2.06 mm^3^ to 48.70 ± 6.88 mm^3^, 86.56 ± 7.72 mm^3^, 123.80 ± 12.18 mm^3^ and 146.94 ± 15.28 mm^3^ at days 0, 3, 6, 9 and 12, respectively. Notably, tumor volumes were significantly smaller in SHED-Exos treated tumors compared with the PBS treated tumors (Fig. 5D, E). Next, tumor tissues were harvested and examined by microscopy at 3 days after the fourth injection of SHED-Exos. The number of micro-vessels surrounding the tumors was reduced in the SHED-Exos-treated group (Fig. 5F, G). To further confirm the decreased micro-vascular formation in the SHED-Exos-treated tumors, immunohistochemical staining for CD31 was performed. The results revealed that OSCCs injected with SHED-Exos produced less vasculature around the base of the tumor and had a lower percentage of CD31-positive cells than the PBS-treated group (Fig. 5H, I). Taking these results into account, we confirmed that SHED-Exos suppress the in vivo micro-vascular formation in OSCC tumors, which can potentially play a crucial role in anti-tumor function.

### SHED-Exos transport both miR-100-5p and miR-1246 to target VEGFA to inhibit the tube formation of endothelial cells

**Fig. 6:**
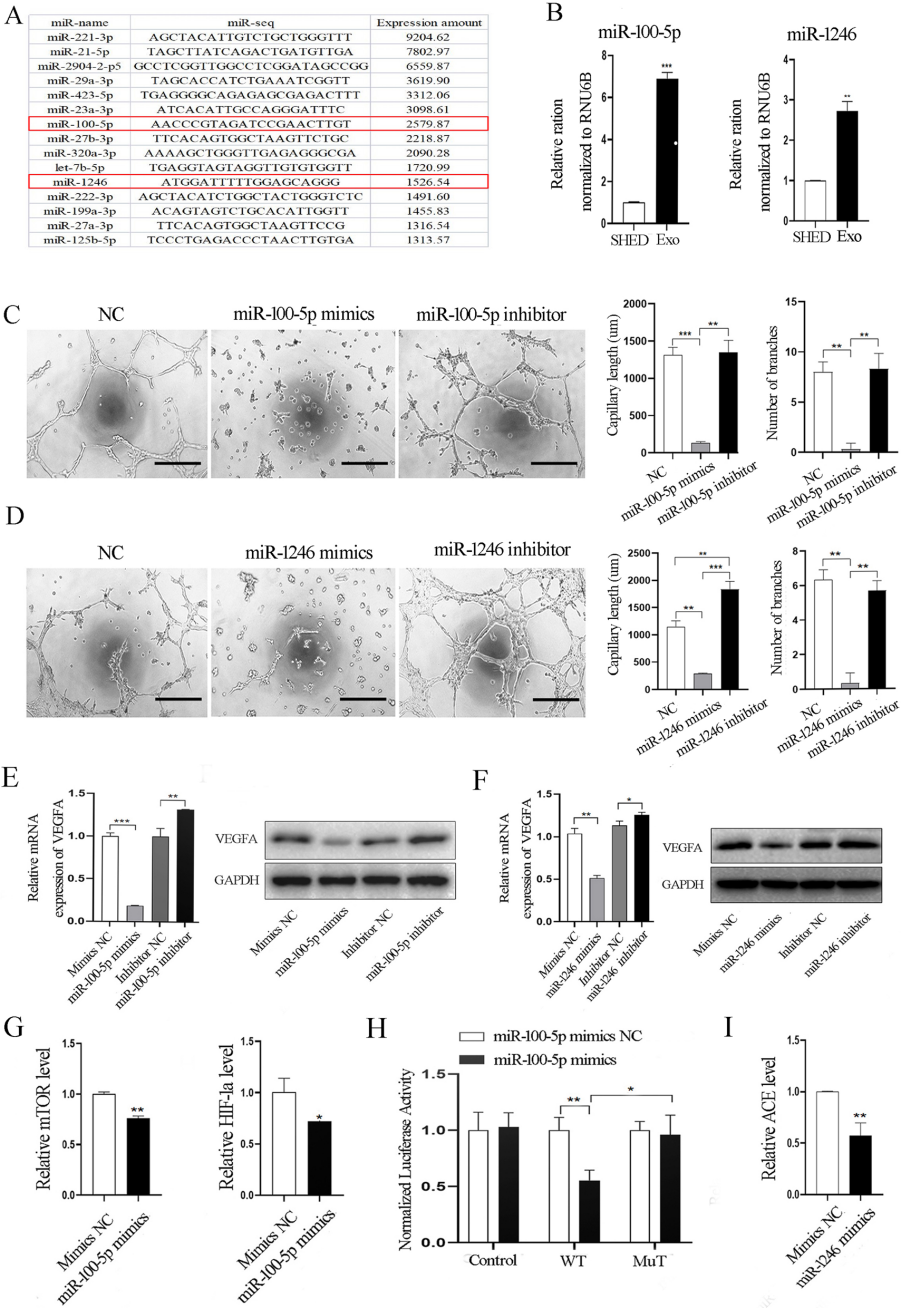
SHED-Exos transport miR-100-5p and miR-1246 to target VEGFA that inhibits endothelial cell tube formation. **A** The top 15 highly expressed miRNAs according to miRNA sequencing. **B** qRT-PCR analysis validating the relative ratio of the expression levels of miR-100-5p and miR-1246 in SHED-Exos and in SHED cells. **C** left, Matrigel tube formation assay of mimics, inhibitor and NC of miR-100-5p and the tube length and branches number in the network structures of endothelial cells; right, quantification of results shown on the left. **D** left, Matrigel tube formation assay of mimics, inhibitor and NC of miR-1246 and the tube length and branches number in the network structures of endothelial cells; right, quantification of results shown on the left. **E** The expression of VEGFA was validated in groups of mimics, mimics NC, inhibitor and inhibitor NC of miR-100-5p by qRT-PCR and Western blot. **F** The expression of VEGFA was validated in groups of mimics, mimics NC, inhibitor and inhibitor NC of miR-1246 by qRT-PCR and Western blot. **G** The mTOR and HIF-1a expression levels were analyzed by qRT-PCR in endothelial cells transfected with miR-100-5p mimics and mimics NC.** H** The target relationship between miR-100-5p and mTOR was verified using a dual-luciferase reporter assay in 293 T cells. **I** The ACE expression level was analyzed by qRT-PCR in endothelial cells transfected with miR-1246 mimics and mimics NC. All experiments were performed three times, and error bars represent means ± SD; P values are indicated with “*”, * indicates P < 0.05, ** indicates P < 0.01, *** indicates P < 0.001

To understand the potential molecular mechanism involved in how exosomes regulate the tube formation of endothelial cells, we performed miRNA sequencing to evaluate the expression profiles of miRNAs encapsulated in SHED-Exos since exosomal miRNAs have been shown to play a role as essential mediators for intercellular communications. According to the expression levels, which are defined as the normalized data of the number of reads for each isoform at the 5' or 3' end of the corresponding miRNA, the top 15 highly expressed miRNAs were identified (as listed in Fig. 6A), which include miR-221-3p, miR-21-5p, miR-2904–2-p5, miR-29a-3p, miR-423-5p, miR-23a-3p, miR-100-5p, miR-27b-3p, miR-320a-3p, let-7b-5p, miR-1246, miR-222-3p, miR-199a-3p, miR-27a-3p and miR-125b-5p. We examined those miRNAs in the literature, and found that miR-100-5p and miR-1246 have been reported in previous studies to be enriched in MSCs exosomes [32, 33] and importantly, both miRNAs have been shown to be involved in the angiogenesis process [17, 34]. Therefore, we focused on those two miRNAs in the present study, and our qRT-PCR analysis verified the expression levels of these two selected miRNAs and confirmed that both are enriched in SHED-Exos compared with SHED cells (Fig. 6B).

We then evaluated whether those two miRNAs could alter the ability of tube formation by endothelial cells by transfecting specific mimics and inhibitors of those two miRNAs into HUVECs and then assessing vascular tube formation in vitro. Significantly shorter tubes and fewer branches, nodes and junctions of network structures were observed in the mimics group of miR-100-5p and an evident trend of an enhancing effect on tube formation was observed in the miR-100-5p inhibitor group (Fig. 6C, Additional file 1: Fig. S3). Similar results were found when this experiment was performed using HUVECs transfected with miR-1246 mimics or inhibitor (Fig. 6D, Additional file 1: Fig. S4). Taken together, these data demonstrate that miR-100-5p and miR-1246 are enriched in SHED-Exos and can inhibit the tube formation ability of endothelial cells.

Next, we investigated how those two miRNAs regulate angiogenesis. VEGFA, the most well-known member of the VEGF family, has been shown to be a crucial modulator of vascular permeability and angiogenesis [35, 36]. We transfected specific mimics and inhibitors of the two miRNAs into endothelial cells. qRT-PCR analysis showed that the expression levels of miR-100-5p and miR-1246 in endothelial cells were significantly overexpressed following pretreatment of endothelial cells with the corresponding mimics, but in contrast, expression levels of miR-100-5p and miR-1246 in endothelial cells were suppressed using the specific inhibitor (Additional file 1: Fig. S5). Next, analysis of the mRNA and protein expression levels of VEGFA in endothelial cells transfected with mimics, inhibitor or the corresponding NC of miR-100-5p or miR-1246 showed that the expression of VEGFA was markedly increased in endothelial cells transfected with inhibitor of miR-100-5p or miR-1246, but was significantly decreased in endothelial cells transfected with corresponding miRNA mimics compared with the NC group (Fig. 6E, F and Additional file 1: Fig. S6).

Next, we further explore how both miR-100-5p and miR-1246 regulate VEGFA expression. Both the miRbase (www.mirbase.org) and the Targetscan (www.targetscan.org) databases were used to predict potential targets of miR-100-5p or miR-1246, but no potential binding site of either miR-100-5p or miR-1246 was found in the 3’-UTR region of VEGFA mRNA. It has been reported that miR-100 could directly target mTOR to control VEGFA expression through the mTOR/HIF-1 signaling pathway in endothelial cells in previous studies [37, 38]. qRT-PCR analysis was performed and showed that the expression levels of mTOR and HIF-1 were significantly decreased following pretreatment of endothelial cells with the miR-100-5p mimics (Fig. 6G). The direct target relationship between miR-100-5p and mTOR was further verified using a dual-luciferase reporter assay, which showed that miR-100-5p mimics significantly decreased the luciferase activity of the WT mTOR 3'-UTR construct, but had no significant effect on the mTOR 3'-UTR-MuT reporter (Fig. 6H), suggesting that miR-100-5p regulates VEGFA expression through the mTOR/HIF-1 pathway. Moreover, no binding site of miR-1246 was found in the mTOR 3’-UTR. We analyzed the expression level of ACE in endothelial cells treated with miR-1246 mimics since the miR-1246/ACE signal pathway was reported to regulate VEGFA expression in endothelial cells [34, 39]. We observed that miR-1246 mimics significantly decreased ACE expression in endothelial cells (Fig. 6I). Taking these data together, it indicates that both miR-100-5p and miR-1246 are enriched in SHED-Exos and can inhibit angiogenesis of endothelial cells by indirectly targeting VEGFA expression, likely through the miR-100-5p/mTOR/HIF-1α and/or the miR-1246/ACE pathway.

## Discussion

The present study observed that SHED-Exos inhibit the growth and migration of endothelial cells. Proliferation, viability and migration behaviors are considered to be biological actions required for the recruitment of adjacent endothelial cells at the beginning of capillary sprouting in neovascularization. Meanwhile, we also found that SHED-Exos induce endothelial cell death. The induction of endothelial cell apoptosis may also contribute to the inhibition of angiogenesis, which has been reported previously [40]. For instance, thrombospondin-1, a natural inhibitor of angiogenesis, can induce endothelial cell apoptosis by activating the caspase 3 pathway [41]. It has been reported that VEGFA contributes to inhibiting apoptosis and also to stimulating the proliferation and migration of endothelial cells, followed by the collapse of blood vessels and tumor regression [40]. Therefore, we consider that suppressing endothelial cell growth and migration together with inducing apoptosis by SHED-Exos will lead to inhibiting the tube formation of endothelial cells in vitro*.*

To provide a more credible proof of the suppressive effect of SHED-Exos on micro-vascular formation, we examined the effects of exosomes on the vasculature using the CAM assay and an OSSC xenogeneic transplantation model, both of which demonstrated that SHED-Exos exerted an inhibitory effect on micro-vessel formation in vivo. Importantly, reduced tumor growth was also observed in the SHED-Exos treated group, which was similar to the findings of a previous study [42]. Based on this finding, we speculate that the weaker angiogenesis resulting from SHED-Exos may contribute to inhibiting the growth of tumor cells. Apart from the indirect effect on tumor growth, we reasoned that the direct interaction of SHED-Exos with tumor cells exists via other signaling molecules and factors. The anti-tumor function by SHED-Exos, which may potentially be developed as a new therapeutic strategy for clinical application, needs further study in the future.

Exosomes encapsulated with diverse bioactive cargoes, including miRNAs, are transferred to endothelial cells to regulate cell function and angiogenesis processes [43, 44]. Therefore, we examined the expression profiles of miRNAs in SHED-Exos and identified the top 15 highly expressed miRNAs. After reviewing the literature, we decided to focus on miR-100-5p and miR-1246 in the present study, because they have been reported to be enriched in MSCs-derived exosomes [32, 33] and also to be involved in the angiogenesis process [17, 34]. Importantly, miR-1246 and miR-100-5p were shown to rank as the first and second most abundant miRNAs in SHED-Exos in a previous study by Luo et al. [33]. Of course, we cannot exclude the potential contributions of other miRNAs from SHED-Exos to inhibit angiogenesis, which needs to be further investigated in the future.

To understand how miR-100-5p and miR-1246 suppress angiogenesis, we found that both miR-100-5p and miR-1246 can downregulate VEGFA expression in endothelial cells. However, we did not find any potential binding sites of miR-100-5p or miR-1246 in the 3’ UTR of VEGFA mRNA, indicating that both miR-100-5p and miR-1246 did not directly target VEGFA to control its expression. As reported, miR-100-5p inhibits angiogenesis by suppressing mTOR/HIF-1α/VEGF signaling in breast cancer cells [17], which was also validated in the endothelial cells in the present study. Although diverse roles of miR-1246 in angiogenic activity were also revealed [45, 46], for example, the miR-1246/ACE signal pathway was reported to be involved in the corneal neovascularization and targeted VEGFA expression via the ACE-Ang I-VEGF pathway [34, 39]. In agreement with that report, the present study also confirmed that miR-1246 can positively control ACE expression. Taking these findings into account, we conclude that miR-100-5p and miR-1246, which are enriched in SHED-Exos, target VEGFA, the pivotal direct angiogenic stimulator, to suppress vascular formation by endothelial cells [47].

During the preparation of our manuscript, we found a very recent study, which reported that exosomes derived from SHED aggregates actually enhanced angiogenesis through shuttling miR-26a [48], which is contrary to our finding. After carefully studying the report from Wu et al., we found that the main differences between our study and theirs are the culture methods and the conditions used to prepare SHED-Exos. In the study of Wu et al., the exosomes were produced from SHED aggregate/sheets, which were obtained by adding 100 μg/ml vitamin C and incubating them for 10 days [49, 50], while we used normal culture conditions (i.e., two-dimension culture) to culture SHED cells. The thicker SHED aggregate/sheets containing high-density stem cells may produce exosomal cargoes with different components, such as miRNAs, compared to our normally cultured SHED cells. This contradictory finding further supports the dual effects of MSCs-derived exosomes on angiogenesis that have been reported in recent years. For example, some studies have reported that MSCs-derived exosomes enhance angiogenesis in tissue ischemia diseases [51–53], while other research studies have suggested that exosomes derived from MSCs inhibit the formation of blood vessels [17, 42, 54]. These conflicting results are likely to be due to the varying exosomal molecular cargoes derived from the variability of the forms of donor cells used, the different cell culture conditions, the diverse animal models and different intervals or dosages of exosomal treatments [55]. Therefore, future work will be targeted at understanding how cells produce different types of exosomes under different conditions, which will definitely benefit the clinical applications of exosomes.

Taken together, our study revealed that SHED-Exos inhibit the angiogenic function of endothelial cells in vitro and in vivo*,* mainly through their two miRNA components: miR-100-5p and miR-1246.

## Conclusions

The present study demonstrates that exosomes derived from stem cells of human deciduous exfoliated teeth inhibit proliferation and migration and induce the apoptosis of endothelial cells and significantly suppress micro-vascular formation, which results in an anti-tumor effect on OSSC tumors. The inhibition of angiogenesis by SHED-Exos is mainly through transferring miR-100-5p and miR-1246 to target VEGFA of endothelial cells (shown schematically in Fig, S7). Our study potentially provides a new perspective for anti-angiogenic therapy by SHED-Exos for anti-cancer treatment.

## Supplementary Information

**Additional file 1.** Supplemental Figures and Table.

## Data Availability

The data set used and/or analyzed during the current study is available from the corresponding author upon reasonable request.

## Abbreviations

MSCs: Mesenchymal stem cells
SHED: Stem cells of human deciduous exfoliated teeth
miRNA: MicroRNA
DMEM: Dulbecco’s Modified Eagle Medium
FBS: Fetal bovine serum
CM: Conditioned medium
SHED-Exos: Exosomes derived from SHED
RNU6B: U6B small nuclear RNA
FACS: Flow cytometry
CCK8 assay: Cell Counting Kit-8 assay
ECS: Exosomes concentration solution
BCA: Bicinchoninic acid
TEM: Transmission electron microscope
NTA: Nanoparticle tracking analysis
c-Caspase 3: Cleaved Caspase 3
c-Caspase 9: Cleaved Caspase 9
c-PARP: Cleaved PARP
t-Caspase 3: Total Caspase 3
t-Caspase 9: Total Caspase 9
t-PARP: Total PARP
PBS: Phosphate-buffered saline
PFA: Paraformaldehyde
PMSF: Phenylmethylsulfonyl fluoride
PVDF: Polyvinylidene fluoride
RIPA: Radio immunoprecipitation assay
CAM: Chorioallantoic membrane
OSCC: Oral squamous cell carcinoma
