# Supplementary material for: Exosomes derived from stem cells of human deciduous exfoliated teeth inhibit angiogenesis in vivo and in vitro via the transfer of miR-100-5p and miR-1246
Source: Stem Cell Res Ther. 2022 Mar 3;13:89. doi: 10.1186/s13287-022-02764-9 (PMC8895508; doi:10.1186/s13287-022-02764-9)
Supplement: Supplementary file 1 — Additional file 1. Supplemental Figures and Table. [file 13287_2022_2764_MOESM1_ESM.docx]

**Supplemental Figures, Figure Legends and Supplemental Table**


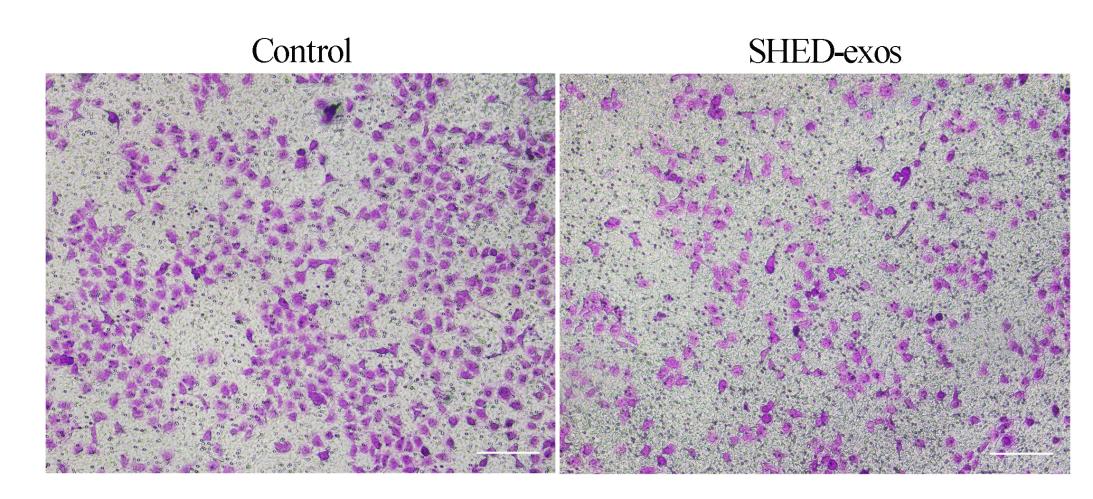


**Fig. S1** **SHED-Exos suppress the migration of endothelial cells**. Transwell migration assays revealing that a significantly lower number of HUVECs migrate through the membrane in the presence of SHED-exosomes compared with PBS. Scale bars =500 μm.


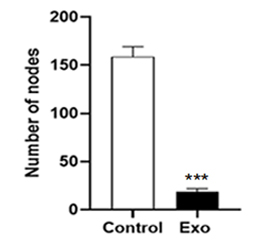

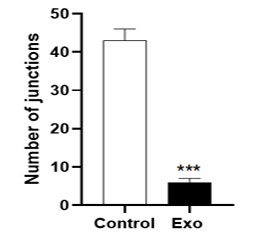


**Fig. S2 SHED-Exos inhibit tube formation by endothelial cells *in vivo***. Quantification of tube nodes and functions in the network structures of HUVECs shown in Fig. 4A. P values are indicated with “*”, *** indicates P < 0.001.


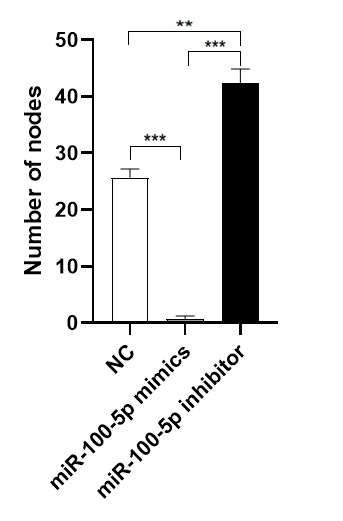

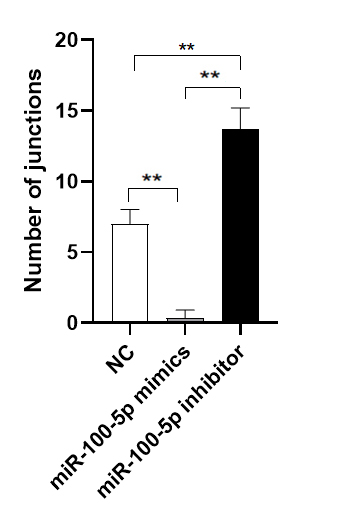


### Fig. S3 Quantification of Matrigel tube formation assay of mimics, inhibitors and NC of miR-100-5p. Quantification of the numbers of tube nodes and functions in the network structures of endothelial cells shown in Fig. 6C. **P < 0.01, ***P < 0.001.


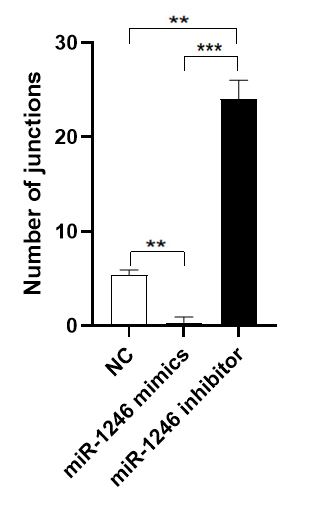

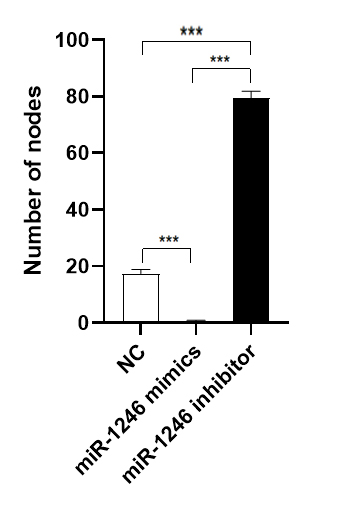


### Fig. S4 Quantification of Matrigel tube formation assays of mimics, inhibitors and NC of miR-1246. Quantification of the numbers of tube nodes and functions in the network structures of endothelial cells shown in Fig. 6D. **P < 0.01, ***P < 0.001.


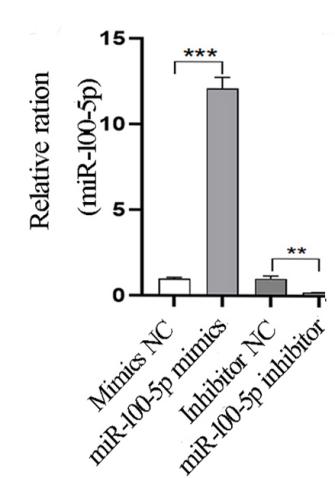

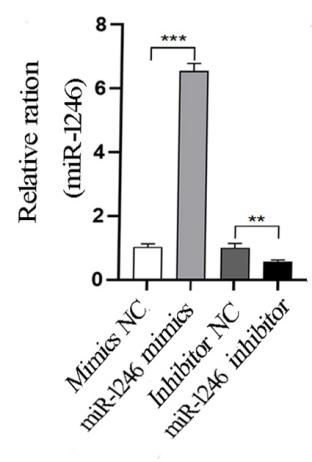


**Fig. S5 The expression of miR-100-5p or miR-1246 was efficiently induced or suppressed by its mimics and inhibitors, respectively.** qRT-PCR analysis validated the relative ratio of expression of miR-100-5p (left panel) and miR-1246 (right panel) in the groups of corresponding mimics NC, mimics, inhibitor NC and inhibitor. P values are indicated with “*”, ** indicates P < 0.01, *** indicates P < 0.001.


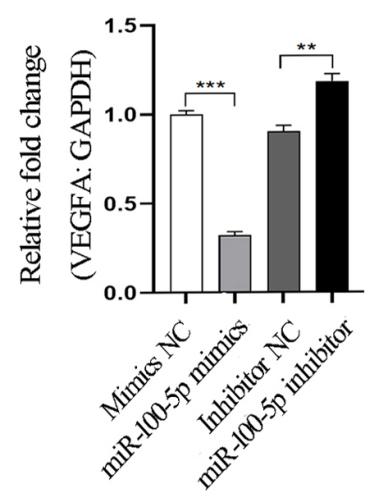

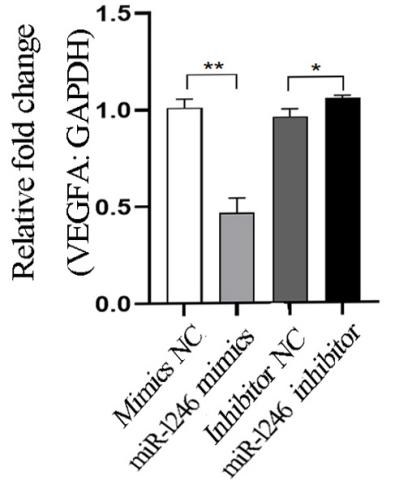


**Fig. S6 Both miR-100-5p and miR-1246 negatively regulate VEGFA protein expression.**

The relative protein level of VEGFA by Western blot in groups of miR-100-5p (left panel) and miR-1246 (right panel) in the corresponding mimics and inhibitor was quantified compared to the respective NC group in Fig. 6E, F. P values are indicated with “*”, * indicates P < 0.05, ** indicates P < 0.01, *** indicates P < 0.001.


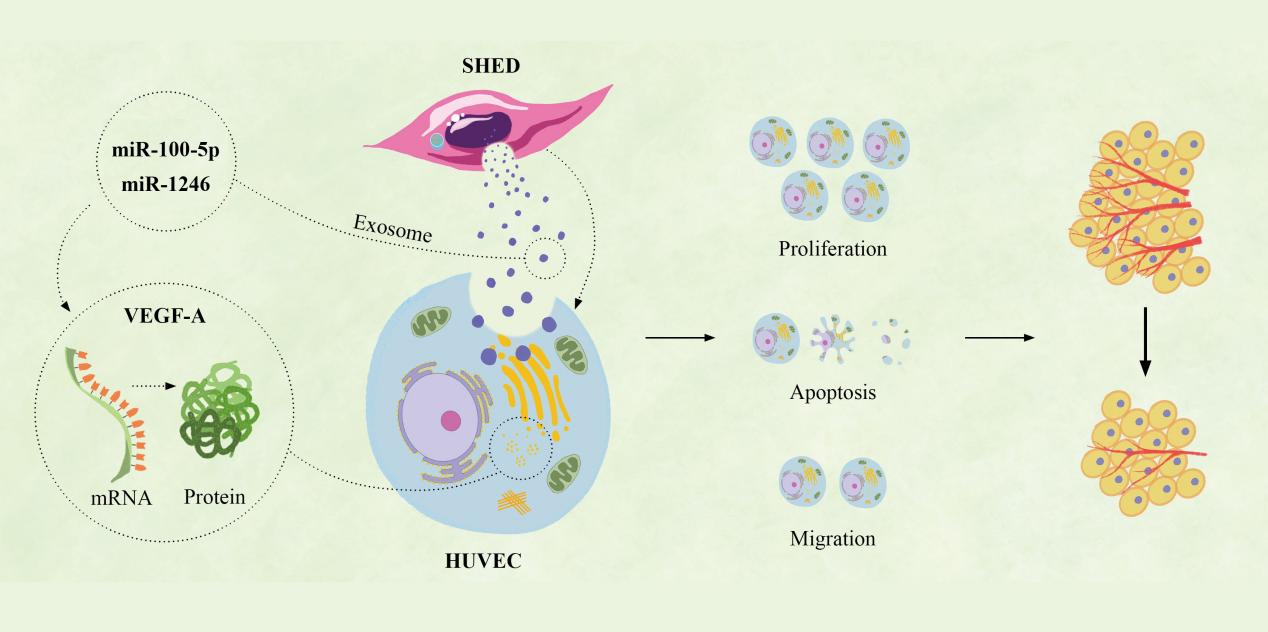


**Fig. S7. Schematic diagram summarizing the results of our study.** SHED-Exos transport miR-100-5p and miR-1246 to target VEGFA of endothelial cells that result in the inhibition of angiogenesis, which could potentially play a crucial role in anti-tumor function.

**Supplementary Table. Oligo sequences used for mRNA qRT-PCR analysis**

| **Gene Name** | **Forward primer** | **Reverse primer** |
| --- | --- | --- |
| GAPDH | ATGACTCTACCCACGGCAAG | GGAAGATGGTGATGGGTTTC |
| VEGFA | GCCCGCTGCTGTCTAATG | TTTACACGTCTCGGATCTTG |
| mTOR | GTTGGCCCTCACCTCAC | CTTGGCAGCTCTCTCACC |
| HIF-1a | GCAGCAACGACACAGAAA | AGCGGTGGGTAATGGAG |
| ACE | TGCAGTTCCAGTTCCATGA | GGCATCCAGGGCATCTAA |
